# Analysis of the trajectory of cognitive function changes and influencing factors in maintenance hemodialysis patients: a prospective longitudinal study

**DOI:** 10.1080/0886022X.2025.2489722

**Published:** 2025-04-14

**Authors:** Wenbin Xu, Xiaolian Long, Yuhe Xiang, Aiyin Yu, Ting Luo, Yuhang Chen, Yan Chen, Qian Yang

**Affiliations:** ^a^ School of Nursing, Chengdu Medical College, Chengdu, Sichuan, China; ^b^ Nursing Department, The Second Affiliated Hospital of Chengdu Medical College, Nuclear Industry 416 Hospital, Chengdu, Sichuan, China; ^c^ General Internal Medicine, Tianfu Campus of Sichuan Cancer Hospital, Chengdu, Sichuan, China; ^d^ Nephrology, The Second Affiliated Hospital of Chengdu Medical College, Nuclear Industry 416 Hospital, Chengdu, Sichuan, China; ^e^ Nursing Department, Sichuan Academy of Medical Sciences, Sichuan Provincial People’s Hospital, Chengdu, Sichuan, China

**Keywords:** Maintenance hemodialysis patients, cognitive function, trajectory of change, latent growth model, influencing factors

## Abstract

**Objectives:**

To explore the trajectory of cognitive function changes and influencing factors in maintenance hemodialysis (MHD) patients.

**Methods:**

A convenience sampling method was used to select MHD patients from a tertiary hospital in Chengdu from August 2023 to April 2024. The general information questionnaire, Chinese version of the Montreal Cognitive Assessment (MoCA), Pittsburgh Sleep Quality Index (PSQI), Appetite Visual Analogue Scale (VAS), and Family Care Index (APGAR) were used for the investigation. Patients’ cognitive function levels were assessed at baseline and at 3, 6, and 9 months after the initial survey. A latent growth model was used to identify potential categories of cognitive function trajectory, and univariate and binary logistic regression analyses were performed to analyze the influencing factors.

**Results:**

A total of 154 MHD patients completed the entire study. The trajectory of cognitive function changes was divided into two potential categories: low cognitive function-fast decline group and high cognitive function-slow decline group. Binary logistic regression results showed that educational level, hypertension, sleep quality, appetite, and family care were influencing factors for the trajectory of cognitive function changes in MHD patients.

**Conclusions:**

Cognitive function in MHD patients showed an overall declining trend over time. The cognitive function change trajectory could be divided into two potential categories: fast decline group and high cognitive function-slow decline group. Healthcare professionals can develop targeted nursing intervention programs based on the characteristics of different patient types and their influencing factors to improve cognitive function and enhance quality of life.

## 1. Background

Chronic kidney disease (CKD) is a common clinical syndrome that often involves multiple systems in the body. Its complex treatment process and less optimistic prognosis have become a serious challenge for global public health systems. Epidemiological surveys show that by 2020, there were 850 million CKD patients worldwide, and China has the largest number of CKD patients globally, with ∼132.3 million, and a prevalence rate of 10.8% [1]. As the disease progresses, kidney function gradually deteriorates, and most CKD patients will eventually develop end-stage renal disease (ESRD), where kidney function is almost entirely lost, necessitating renal replacement therapy to maintain life. Hemodialysis (HD) is the most commonly used renal replacement therapy both domestically and internationally, with about 86% of ESRD patients opting for hemodialysis [2]. Clinically, hemodialysis for ≥3 months is defined as maintenance hemodialysis (MHD). According to the China National Renal Data System (CNRDS), nearly 750,000 hemodialysis patients were registered in mainland China in 2021, and by 2030, the number of ESRD patients is expected to exceed 3 million, with 1.48 million people undergoing dialysis treatment [1], placing severe medical and economic burdens on the country.

Although MHD can effectively alleviate symptoms of end-stage renal failure and extend the survival of patients, it can only replace part of the kidney’s excretory function and cannot completely replace the kidney’s metabolic and endocrine functions. Pathophysiological changes caused by the disease itself and the damage caused during dialysis procedures are still unavoidable [3]. Among these, cognitive impairment is one of the more common damages. Cognitive function refers to the process by which the human brain processes, stores, and retrieves information, essential for executing various social activities, composed of multiple domains, including memory, comprehension, language, orientation, and executive function. Clinically, impairment in two or more functional domains is termed cognitive impairment [4]. Studies show that the prevalence of cognitive impairment in MHD patients ranges from 24 to 67% [5–7]. Numerous studies have also found that cognitive impairment may reduce patients’ adherence to medication and treatment, affect daily life, cause emotional distress, increase the risk of hip fractures, and significantly raise mortality rates [6,7]. As an important indicator of health status, cognitive function directly impacts an individual’s ability to perform daily activities and quality of life. If not promptly prevented or controlled, it can impose significant economic and psychological burdens on caregivers and society. Therefore, preventing or delaying cognitive impairment in MHD patients and improving their quality of life is imperative.

However, there are still many deficiencies in the current research on the cognitive function of Chinese MHD patients. Firstly, although previous studies have shown that MHD patients have a relatively high prevalence of cognitive impairment, the specific trajectory of cognitive function changes and its influencing factors remain unclear. Cognitive function is a dynamically evolving process, and its complexity and variability require in-depth and detailed research methods to comprehensively reveal its changing laws. However, through a systematic review of relevant domestic and foreign literatures, it is found that most of the current research on the cognitive function of MHD patients focuses on cross-sectional surveys. Although this provides a large amount of valuable information, it can only provide the average cognitive function level change at a certain time point or in a certain group, lacking the perspective of deeply exploring the dynamic changes of cognitive function within individuals and groups from the time dimension. In addition, the chronic disease trajectory theoretical model clearly points out [8] that there are significant individual differences in the disease development trajectory and heterogeneity is also shown at the group level, that is, the disease progression paths of different patient groups or different patients within the same group may be very different. In view of the limitations of the above cognitive function research and the heterogeneity of chronic disease trajectories, in-depth research on the change trajectory of cognitive function and its influencing factors in Chinese MHD patients is of great significance for accurately identifying high-risk patient groups and formulating personalized intervention measures. In addition, the special social and cultural background and medical environment faced by Chinese MHD patients also make the research on cognitive function have unique value and urgency. At the same time, with the intensification of population aging in China, the number of elderly MHD patients is constantly increasing, and the risk of cognitive dysfunction also increases accordingly. Therefore, conducting research on the change trajectory of cognitive function in Chinese MHD patients will not only help to fill the gaps in relevant domestic research but also provide more accurate and personalized scientific basis for clinical medical staff, enabling them to formulate more targeted intervention measures according to the unique cognitive function change trajectory of each patient, thus effectively preventing or delaying the occurrence of cognitive impairment in MHD patients, improving the quality of life of patients, and reducing the social burden brought about thereby.

Based on this, this study adopts a prospective longitudinal research method and tracks the change trajectory of cognitive function in MHD patients based on the latent growth model (LGM), deeply analyzing the potential factors influencing the formation of these trajectory categories, to provide more accurate and personalized scientific basis for clinical medical staff, enabling them to formulate more targeted intervention measures according to the unique cognitive function change trajectory of each patient, thus effectively preventing or delaying the occurrence of cognitive impairment in MHD patients, improving the quality of life of patients, and reducing the social burden brought about thereby.

## 2. Subjects and methods

### 2.1. Research subjects

This study adopted a convenience sampling method, selecting patients receiving maintenance hemodialysis from the hemodialysis center of a tertiary hospital in Chengdu from August 2023 to April 2024 as the research subjects. Inclusion criteria: patients diagnosed with end-stage renal disease according to the Kidney Disease Outcomes Quality Initiative (K/DOQI) [9] guidelines and receiving regular hemodialysis for ≥3 months; no history of cerebrovascular accidents, neurological, or psychiatric disorders; age ≥18 years, able to communicate normally, and consent to the study. Exclusion criteria: patients with hyperthyroidism, tuberculosis, cancer, or other organ failure; patients with organic mental disorders; patients who developed other serious illnesses or died during the study; and those who refused follow-up or voluntarily withdrew from the study.

### 2.2. Research tools

#### 2.2.1. General Information Questionnaire

The research team first constructed an item pool through extensive literature reviews, and then developed it through expert consultations and multiple team discussions, It has a certain degree of scientificity. the questionnaire includes items on biologic sex, age, educational level, living conditions, exercise habits, monthly per capita household income, economic satisfaction, hypertension, diabetes, and smartphone usage.

#### 2.2.2. Chinese version of the Montreal Cognitive Assessment (MoCA)

This scale is used to assess the cognitive function of patients. It was developed by Nasreddine et al. [10] in 2004 based on the Mini-Mental State Examination (MMSE) and was translated into Chinese by Zhang [11]. The MoCA is specifically designed for mild cognitive impairment (MCI) and has been widely used internationally. It includes 7 cognitive domains and a total of 11 test items. The total score is 30 points, with a score below 26 indicating cognitive impairment. If the subject’s education level is <12 years, one additional point is added. The reliability and validity of this scale have been confirmed in CKD patients, with a Cronbach’s *α* coefficient of 0.818.

#### 2.2.3. Pittsburgh Sleep Quality Index (PSQI)

This scale is used to assess sleep quality over the past month. It was developed by Buysse et al. [12] in 1989 and translated into Chinese by Lu [13]. The scale includes 7 components (18 items in total), with a total score range of 0–21. Scores between 8 and 11 indicate mild sleep disturbances, 12–16 indicate moderate sleep disturbances, and scores above 17 indicate severe sleep disturbances. The Cronbach’s *α* coefficient for this scale is 0.83.

#### 2.2.4. Appetite Visual Analogue Scale (VAS)

This scale is used for the subjective assessment of appetite in maintenance hemodialysis patients. It was developed by Molfino et al. [14] in 2016. The scale uses a 100 mm line, where each millimeter represents 1 point, with 0 indicating ‘no appetite at all’ and 100 indicating ‘very good appetite’. The patient marks a point representing their appetite perception over the past week, with higher scores indicating better appetite. A score of ≤60 points indicates reduced appetite. This scale is easy to use, highly sensitive, and has good reliability and validity.

#### 2.2.5. Family Care Index Questionnaire (APGAR)

This scale is used to assess the degree of family care, developed by Smilkstein et al. [15] in 1978 and translated into Chinese by Lv [16]. The scale consists of 5 items, representing adaptability, intimacy, growth, cooperation, and affection. It uses a three-level scoring method, with 0–2 points assigned from ‘rarely’ to ‘often’. A total score of 0–3 indicates severe family dysfunction, 4–6 indicates moderate dysfunction, and 7–10 indicates good family functioning. The Cronbach’s *α* coefficient for this scale is 0.911.

### 2.3. Sample size

The sample size for this study was calculated based on the method for estimating sample size in multivariate analysis [17], which requires at least 5–10 times the number of variables. There were 26 variables in this study (10 items in the general information questionnaire, 7 dimensions in the MoCA, 7 dimensions in the PSQI, 1 dimension in the VAS, and 1 dimension in the APGAR). Considering a loss-to-follow-up rate of 5–10%, the sample size range was determined to be 136–275 cases. A total of 154 patients were included in the study.

### 2.4. Investigation methods and quality control

This study was conducted by members of the research team who had received standardized training. Data collection and follow-up were carried out *via* questionnaire surveys. There were four survey time points: baseline (T0), which was at the time of patient enrollment, and follow-ups at 3 months (T1), 6 months (T2), and 9 months (T3) after the initial survey. At T0, the research team conducted a detailed data collection using the general information questionnaire, MoCA, PSQI, VAS, and APGAR through face-to-face interviews. At T1, T2, and T3, follow-up surveys of cognitive function were conducted by telephone using the MoCA. To ensure the objectivity and accuracy of the study results and avoid common method bias caused by the use of the same measurement tools, Harman’s single-factor test was used to check for common method bias.

It is worth mentioning that in this study, the use of MOCA strictly followed the guidelines of the scale. Through rigorous research design and preliminary exploratory analysis, we determined to conduct the scale assessment at least 24 h after the end of dialysis at each follow-up time point [18]. This is based on previous studies showing that the dialysis process may have an immediate impact on the patient’s cognitive state due to factors, such as hemodynamic changes and electrolyte fluctuations. However, an interval of 24 h can make the patient’s physical state relatively stable, minimizing the interference of dialysis operations on cognitive function assessment, thus more accurately reflecting the patient’s cognitive level in a daily stable state. In addition, for the information collection of MOCA, we formed a professional research team. All personnel involved in the scale assessment received strict and systematic training. The training content covered multiple aspects, such as the theoretical basis of MOCA, assessment rules, operation procedures, and coping strategies for common problems. After the training, multiple mock assessments and actual case assessments were carried out to ensure that each administrator’s understanding depth, application proficiency, and scoring consistency of the scale reached a high standard. This ensured the standardized use of the MOCA scale throughout the research process and effectively improved the quality and reliability of data collection.

### 2.5. Statistical methods

IBM SPSS 26.0 and IBM Mplus 8.3 software were used for statistical analysis. Normally distributed measurement data were described by mean ± standard deviation, while skewed data were described by median and quartiles. Count data were represented by frequency and percentage. Mplus 8.3 software was used to build the latent growth model (LGM), and its goodness-of-fit was evaluated to analyze whether there was heterogeneity in the cognitive function trajectory of MHD patients. Three criteria were used to estimate the optimal model for different combinations: (1) The smaller the Akaike Information Criterion (AIC), Bayesian Information Criterion (BIC), and adjusted BIC (aBIC), the better the model fit; (2) The Lo-Mendell-Rubin likelihood ratio test (LMRT) and Bootstrap likelihood ratio test (BLRT), where statistical significance (*p* < 0.05) indicates that the k-class model is more suitable than the k-1 class model; (3) The closer the information entropy (Entropy) is to 1, the more accurate the classification. When Entropy = 0.8, it indicates that the classification accuracy of the model is >90% [19,20]. In the univariate analysis, normally distributed data with homogeneity of variance were analyzed by analysis of variance (ANOVA), chi-square test, or Fisher’s exact test; otherwise, non-parametric tests were used. For multivariate analysis, binary logistic regression was used with the potential class as the dependent variable, and the variables with statistical significance in the univariate analysis were used as covariates to further analyze the influencing factors. A *p*-value of <0.05 was considered statistically significant.

## 3. Results

### 3.1. General data of the study participants

**Figure 1.**
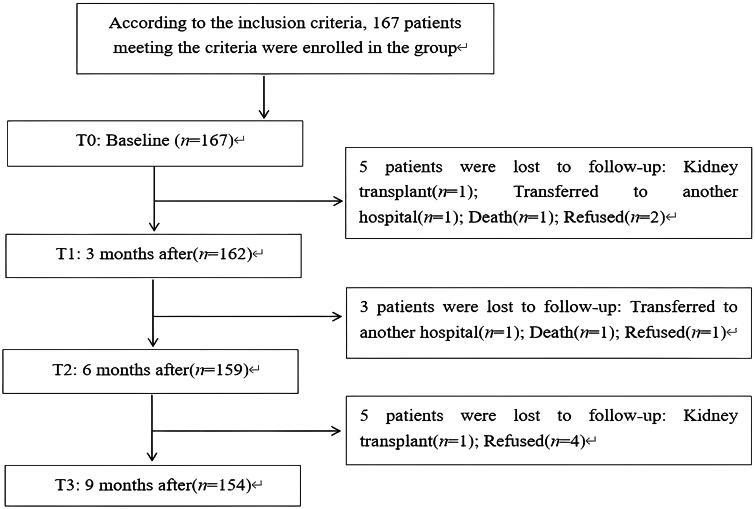
Patient enrollment flow chart.

A total of 167 MHD patients were enrolled in the baseline survey (T0). During the 9-month follow-up, 5 patients were lost to follow-up at T1 (after 3 months), 3 patients at T2 (after 6 months), and 5 patients at T3 (after 9 months). Ultimately, 154 patients completed the entire study, with a loss to follow-up rate of 7.8%. The specific content can be seen in the patient enrollment flow chart (Figure 1). Among the 154 patients included in the study, 91 (59%) were male, most patients were under 65 years old (71%), more than half had a junior high school education or below (61%), almost all lived with family members (96%), and 125 patients felt that their economic satisfaction was insufficient (81%). See Table 1 for specific details.

**Table 1.** General information of the study subjects (*n* = 154).

Variables	Category	Number	Composition ration (%)
Sex	Male	91	59
	Female	63	41
Age	≤65 years	110	71
	>65 years	44	29
Education level	Junior high or below	94	61
	High school or above	60	39
Exercise frequency	Never or occasionally	78	51
	Often	76	49
Living situation	With family	147	95
	Alone	7	5
Per capita monthly income	<5000 Yuan	50	33
	≥5000 Yuan	104	68
Economic satisfaction	Insufficient	125	81
	Sufficient	29	19
Diabetes	Yes	100	65
	No	54	35
Hypertension	Yes	94	61
	No	60	39
Smartphone usage	Yes	137	89
	No	17	11

### 3.2. Common method bias testing

Harman’s single-factor test was used to test for common method bias in the four rounds of data. The results of the unrotated exploratory factor analysis showed that there were 8, 9, 8, and 7 factors with eigenvalues >1 in the four measurements, respectively, and the variance explained by the first factor was 27.50, 21.14, 26.41, and 30.95%, all of which were below the critical value of 40.00%. Therefore, this study does not have a significant common method bias problem [21].

### 3.3. Identifying the latent classes of cognitive function trajectories in MHD patients

The heterogeneity of cognitive function scores for 154 MHD patients who completed all four rounds of surveys was analyzed, and the LGM model was used for model fitting. The specific results are shown in Table 2. Starting with Model 1, the number of classes in the model was gradually increased, and a total of 1–4 classes were fitted. The AIC, BIC, and aBIC values all gradually decreased with the increase in the number of classes, indicating that the more classes, the better the model fit. However, the LMRT result showed no statistical significance starting from the fourth class (*p* > 0.05), indicating that further increasing the number of classes did not bring significant statistical improvements. Furthermore, the Entropy value peaked at two classes and then gradually decreased, suggesting that the model fit worsened starting from the third class. Therefore, based on a comprehensive comparison of the fitting results, Model 2 was selected as the best-fitting model. Additionally, the average class membership probabilities for the two latent classes were 94.10 and 97.30%, respectively, further confirming the model’s high stability and reliability in classification. See Table 3 for details.

**Table 2.** Model fit results for the latent classes of cognitive function trajectories in MHD patients.

Category numbers	AIC	BIC	aBIC	*p*		Entropy	Category ration
				LMRT	BLRT		
1	3717.629	3735.851	3716.86	–	–	–	1
**2**	**3591.806**	**3619.139**	**3590.652**	**0.0011**	**0**	**0.891**	**0.260/0.740**
3	3563.261	3599.704	3561.722	0.0454	0	0.83	0.676/0.091/0.233
4	3538.236	3583.791	3536.313	0.2026	0	0.849	0.222/0.084/0.584/0.110

AIC: Akaike Information Criterion; BIC: Bayesian Information Criterion; aBIC: adjusted BIC; LMRT: Lo-Mendell-Rubin likelihood ratio Test; BLRT: Bootstrap Likelihood Ratio Test; *p*: *p*-value.

The bolded values represent the potential categories selected in this study.

**Table 3.** Average class membership probabilities for two latent classes in MHD patients (row for each class).

Class	C1 (%)	C2 (%)
C1	94.10	5.90
C2	2.70	97.30

### 3.4. Naming and determining the subgroups of cognitive function trajectories in MHD patients

**Figure 2.**
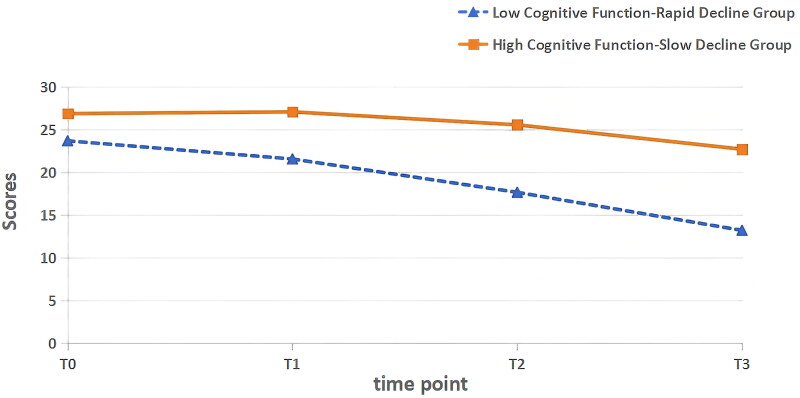
Trajectory of cognitive function changes in MHD patients.

Based on the results of the latent class analysis, a trajectory diagram was drawn, with the cognitive function scores of MHD patients on the vertical axis and the time points from T0 to T3 on the horizontal axis, as shown in Figure 2. Based on the initial levels and rates of change, the two categories were named as follows: (1) C1: The average initial cognitive function score of the C1 group was (23.68 ± 5.274), and the intercept was 24.444, indicating that the cognitive function of the C1 group was poor at T0. Cognitive function showed a rapid decline during the follow-up (slope = −3.460, *p* < 0.001), hence this group was named the ‘Low Cognitive Function-Rapid Decline Group’, with 40 patients (26.0%). (2) C2: The average initial cognitive function score of the C2 group was (26.86 ± 3.931), and the intercept was 27.770, indicating that the cognitive function of the C2 group was better at T0. During the follow-up, cognitive function showed a slow decline (slope = −1.400, *p* < 0.001), hence this group was named the ‘High Cognitive Function-Slow Decline Group’, with 114 patients (74.0%). A comparison of cognitive function scores at different time points between the two groups is shown in Table 4.

**Table 4.** Comparison of cognitive function scores at different time points between the two groups (*n* = 154).

Group	Number	T0	T1	T2	T3
C1	40	23.68 ± 5.274	21.55 ± 4.782	17.65 ± 2.723	13.20 ± 2.298
C2	114	26.86 ± 3.931	27.06 ± 3.543	25.54 ± 3.224	22.69 ± 4.228
*F*	–	16.127	59.171	191.650	164.165
*p*	–	**<0.001**	**<0.001**	**<0.001**	**<0.001**

C1: low cognitive function-rapid decline group; C2: high cognitive function-slow decline group; *p*: *p*-value; *F*: variance analysis.

The bolded values represent statistically significant differences.

### 3.5. Univariate analysis of the latent classes of cognitive function trajectories in MHD patients

The two latent classes of cognitive function trajectories were used as dependent variables, and the demographic data, APGAR scores, VAS scores, and PSQI scores were used as independent variables for univariate analysis. The results showed statistically significant differences between the two categories in terms of age, educational level, hypertension, diabetes, smartphone usage, APGAR scores, VAS scores, and PSQI scores (*p* < 0.05). See Table 5 for specific results.

**Table 5.** Univariate analysis of the latent classes of cognitive function trajectories in MHD patients (*n* = 154).

Variables	Category	Low cognitive function-rapid decline group (*n* = 40)	High cognitive function-slow decline group (*n* = 114)	*χ*^2^/*F*	*p*
Sex [*n* (%)]				0.971^a^	0.324
	Male	21 (52.5%)	70 (61.4%)		
	Female	19 (47.5%)	44 (38.6%)		
Age [*n* (%)]				5.137^a^	0.023*
	≤65years	23 (57.5%)	87 (76.3%)		
	>65years	17 (42.5%)	27 (23.7%)		
Exercise frequency [*n* (%)]				0.690^a^	0.406
	Never or occasionally	18 (45.0%)	60 (52.6%)		
	Often	22 (55.0%)	54 (47.4%)		
Educational level [*n* (%)]				65.130^a^	<0.001**
	Junior high or below	3 (7.5%)	91 (79.8%)		
	High school or above	37 (92.5%)	23 (20.2%)		
Living situation [*n* (%)]					
	With family	38 (95.0%)	109 (95.6%)	0.260^b^	0.584
	Alone	2 (5.0%)	5 (4.4%)		
Per capita monthly income [*n* (%)]				2.480^b^	0.115
	<5000 Yuan	17 (42.5%)	33 (28.9%)		
	≥5000 Yuan	23 (57.5%)	81 (71.1%)		
Economic Satisfaction [*n* (%)]				0.519^b^	0.471
	Insufficient	34 (85.0%)	91 (79.8%)		
	Sufficient	6 (15.0%)	23 (20.2%)		
Diabetes [*n* (%)]				21.266^a^	<0.001**
	Yes	14 (35.0%)	86 (75.4%)		
	No	26 (65.0%)	28 (24.6%)		
Hypertension [*n* (%)]				65.130^a^	<0.001**
	Yes	3 (7.5%)	91 (79.8%)		
	No	37 (92.5%)	23 (20.2%)		
Smartphone usage [*n* (%)]				31.591^b^	<0.001**
	Yes	26 (65.0%)	111 (97.4%)		
	No	14 (14.0%)	3 (2.6%)		
APGAR (±*S*)	—	6.16 ± 3.01	8.47 ± 1.93	30.639^c^	<0.001**
VAS (±*S*)	—	52.77 ± 16.85	64.04 ± 8.03	31.120^c^	<0.001**
PSQI (±*S*)	—	12.30 ± 5.05	6.87 ± 4.90	35.835^c^	<0.001**

*p*: *p*-value; APGAR: Family Care Index Questionnaire; VAS: Appetite Visual Analogue Scale; PSQI: Pittsburgh Sleep Quality Index.

The bolded values represent statistically significant differences.

*p < 0.05; **p < 0.001.

^a^Chi-square test.

^b^Fisher test.

^c^Variance analysis.

### 3.6. Multivariate analysis of the latent classes of cognitive function trajectories in MHD patients

The potential categories of cognitive function change trajectories were used as the dependent variables (low cognitive function-rapid decline group = 0, high cognitive function-slow decline group = 1), and the statistically significant variables from the univariate analysis were used as independent variables for multivariate binary logistic regression analysis. The specific variable assignments are shown in Table 6. The results show that educational level, hypertension, APGAR score, VAS score, and PSQI score are influencing factors for the trajectory of cognitive function change in MHD patients, and the differences were statistically significant (*p* < 0.05). The results of the multivariate analysis are shown in Table 7.

**Table 6.** Variable assignment for binary logistic regression analysis.

Variables	Coding method
Age	≤65 years = 0, >65 years = 1
Educational level	Junior high or below = 0, High school or above = 1
Hypertension	No = 0, Yes = 1
Diabetes	No = 0, Yes = 1
Smartphone usage	No = 0, Yes = 1
APGAR	Original value
VAS	Original value
PSQI	Original value

APGAR: Family Care Index Questionnaire; VAS: Appetite Visual Analogue Scale; PSQI: Pittsburgh Sleep Quality Index.

**Table 7.** Multivariate analysis of the latent classes of cognitive function Trajectories in MHD patients (*n* = 154).

Variables	*β*	*SE*	Wald *χ*^2^	OR	95%CI	*p*
Age (>65 years)	−0.428	0.929	1.283	0.652	0.129–3.289	0.604
Educational level (high school or above)	−2.605	0.994	6.873	0.74	0.011–0.518	0.009*
Hypertension (yes)	3.150	1.161	7.367	23.345	2.400–227.089	0.007*
Diabetes (yes)	−1.052	0.929	1.283	0.349	0.057–2.157	0.257
Smartphone usage (yes)	2.058	1.290	2.545	7.829	0.625–98.083	0.111
APGAR	0.489	0.157	9.668	1.630	1.198–2.219	0.020*
VAS	0.079	0.031	6.301	1.082	1.017–1.151	0.012*
PSQI	−0.160	0.076	4.477	0.852	0.735–0.988	0.034*
Constant	−6.494	3.090	4.418	0.02	—	0.036

*β*: regression coefficient; *SE*: standard error; Wald *χ*^2^: Wald chi-square value; OR: odds ratio; 95%CI: 95% confidence interval; *p*: *p*-value; APGAR: Family Care Index Questionnaire; VAS: Appetite Visual Analogue Scale; PSQI: Pittsburgh Sleep Quality Index.

The bolded values represent statistically significant differences.

*p < 0.05.

## 4. Discussion

### 4.1. The cognitive function trajectory in MHD patients shows a linear declining trend

The results of this study show that the trajectory of cognitive function in MHD patients presents a downward trend, with MoCA scores generally in the middle-to-lower range, indicating a widespread lack of cognitive function in MHD patients, with a continued deterioration over time. Wang et al. [22] found in a three-year multicenter prospective cohort study that the cognitive function of some maintenance dialysis patients was relatively low and continued to decline over time. It has been reported that since the brain and kidney share similar microvascular structures and hemodynamic fluctuations, they are both susceptible to some common vascular damage risk factors, including inflammation, atherosclerosis, and oxidative stress. These shared factors are considered the important pathological basis for the development of cognitive impairment and dementia in MHD patients [23]. Notably, the cognitive impairment associated with MHD is often insidious and progresses slowly, making it difficult to detect early, thereby delaying intervention. As the disease progresses, patients face not only further physical deterioration but also exacerbated psychological issues. These factors, combined, weaken the body’s immune surveillance and cytotoxic abilities, not only affecting treatment outcomes and prognosis but also aggravating cognitive impairment [5]. Therefore, healthcare workers should strengthen the early screening of cognitive function impairment in MHD patients, targeting modifiable risk factors for early prevention and management to delay the onset and progression of cognitive impairment, improving patients’ adherence to treatment and clinical outcomes, and enhancing their quality of life.

It has been reported that the incidence of cognitive impairment in peritoneal dialysis patients is lower compared to hemodialysis patients [24]. Currently, the specific causes of cognitive impairment in peritoneal dialysis patients remain unclear. This may be related to issues, such as chronic inflammatory states and the accumulation of uremic toxins in peritoneal dialysis patients. These factors can affect the function of the blood–brain barrier, leading to damage to nerve cells and triggering cognitive impairment [25]. However, some studies have shown that hemodialysis patients are prone to problems, such as insufficient cerebral perfusion during dialysis, hemodynamic fluctuations, and arterial stiffness, which may lead to ischemic damage to the cerebrovascular system [26]. In contrast, peritoneal dialysis provides a consistent hemodynamic environment [26], which is theoretically more beneficial for protecting cognitive function. However, there are few longitudinal studies on the cognitive function of dialysis patients. The sample sizes of the conducted studies are relatively small, and there is a large degree of heterogeneity in the studies, which affects the reliability of the results. Different studies vary in aspects, such as the choice of cognitive function assessment tools, the inclusion criteria of study subjects, and dialysis treatment regimens. These differences may mask the true differences in cognitive impairment between hemodialysis and peritoneal dialysis. Future studies with larger sample sizes are needed to further explore this relationship and determine whether peritoneal dialysis may provide a protective effect on cognitive function.

It is worth noting that Ali et al. [27] conducted a systematic review and meta-analysis on the impact of dialysis modality choice on cognitive function in ESRD patients. This study included 11 studies with a total of 195,774 patients. The results indicated that PD might be a more reasonable RRT, as PD patients receive daily treatment with relatively stable molecular changes, better clearance of middle molecules, and more stable hemodynamics, contributing to better preservation of residual kidney function [28]. Additionally, PD patients have greater flexibility in scheduling, which is beneficial for improving overall mental well-being [29]. However, despite sufficient evidence linking cognitive impairment to renal dysfunction, the impact of different RRT modalities on the progression of cognitive impairment remains unclear. Furthermore, this study has certain limitations, such as the cross-sectional nature of the included studies, short follow-up durations, inconsistencies in cognitive impairment assessment tools, variations in dialysis duration, and unclear baseline cognitive function. Future prospective studies should consider cognitive decline as a meaningful outcome measure.

### 4.2. Different trajectories of cognitive function exist in MHD patients

The results of this study show that the cognitive function trajectories of MHD patients can be divided into two categories: low cognitive function-rapid decline group and high cognitive function-slow decline group. (1) Low Cognitive Function-Rapid Decline Group (26.0%): The initial average cognitive function score for this group was (23.68 ± 5.274), indicating poor cognitive function. During the follow-up, cognitive function showed a rapid overall decline, which may be related to the generally older age of patients in this group. Previous studies have shown that advanced age is an independent risk factor for cognitive impairment in MHD patients [23]. The mechanism may involve brain neural network aging, reduced synaptic density, and insufficient cognitive stimulation caused by reduced social activity, which together contribute to the rapid decline in multi-dimensional cognitive abilities, such as memory, executive function, and attention [30]. Therefore, healthcare providers should closely monitor cognitive function changes in this group, particularly among elderly patients, regularly assess cognitive function, and promptly detect and intervene in potential cerebrovascular complications. In addition, personalized health education should be provided to enhance patients’ and their families’ understanding of the disease and self-management abilities, and cognitive training interventions, such as memory strengthening and attention exercises, should be introduced to delay or improve cognitive function deterioration. (2) High Cognitive Function-Slow Decline Group (74.0%): The initial cognitive function score for this group was (26.86 ± 3.931), indicating good cognitive function. During the follow-up, cognitive function showed a slow overall decline. Interestingly, the cognitive function of this group improved during the T1 period. This may be due to the early stage of dialysis when the clearance of toxins, correction of electrolyte imbalances, and acid-base disturbances improved the brain’s microenvironment, resulting in short-term cognitive function improvement [31]. However, as dialysis treatment continues, repeated mechanical operations may exacerbate fatigue and emotional distress in patients. In addition, long-term dialysis can cause severe changes in hemodynamics and blood volume, thereby reducing cognitive function [32]. For this group, healthcare providers should focus on providing psychological support services, helping patients manage their emotions, and maintaining a positive and optimistic mindset. Early screening and preventive management measures should be advocated, encouraging patients to adopt healthy lifestyles, including a balanced diet, regular exercise, adequate sleep, and appropriate cognitive training activities to delay the natural decline of cognitive function as much as possible.

It is worth noting that the results of this study found that in the Low Cognitive Function-Rapid Decline Group, 42.5% of patients were aged >65 years, whereas in the High Cognitive Function-Slow Decline Group, only 23.7% of patients were in this age range. This suggests that the proportion of elderly patients is relatively higher in the rapid decline group. Studies have shown that aging can lead to the aging of neural networks and a decrease in synaptic density, which results in cognitive decline [30], and this may contribute to the differences in cognitive function trajectory between the two groups. Additionally, the average PSQI score of the Low Cognitive Function-Rapid Decline Group was 12.30 ± 5.05, indicating moderate sleep disturbances, while the High Cognitive Function-Slow Decline Group had a score of 6.87 ± 4.90, indicating relatively better sleep quality. Poor sleep quality can impair the brain’s ability to clear toxic molecules and affect neurotransmitter balance [33], thereby damaging cognitive function, which could explain the faster rate of cognitive decline in the Low Cognitive Function-Rapid Decline Group. Regarding appetite, the Low Cognitive Function-Rapid Decline Group had an average VAS score of 52.77 ± 16.85, while the High Cognitive Function-Slow Decline Group had a score of 64.04 ± 8.03, suggesting relatively poorer appetite in the rapid decline group. Maintenance dialysis patients may experience metabolic disturbances that lead to reduced appetite and protein-energy wasting, which can result in malnutrition and affect cognitive function [34], serving as another influencing factor for the differences between the two groups. On the family care index, the Low Cognitive Function-Rapid Decline Group had an average APGAR score of 6.16 ± 3.01, while the High Cognitive Function-Slow Decline Group had a score of 8.47 ± 1.93, indicating that the rapid decline group received less family care. Family support can alleviate negative emotions, encourage participation in cognitive training and social activities, and protect cognitive function. The varying levels of family care further exacerbate the differences in the cognitive function trajectories between the two groups.

### 4.3. Influencing factors of the cognitive function change trajectory in MHD patients

#### 4.3.1. Educational level

The results of this study show that patients with a high school education or above have a 26.0% lower risk of cognitive impairment compared to those with a junior high education or below, indicating that a higher educational level serves as a protective factor for cognitive function in MHD patients (OR = 0.74, *p* = 0.009). This finding is consistent with the results of Brigola et al. [35]. From both biological and psychosocial perspectives, intelligence is influenced not only by genetic factors and brain function but also by educational experience. A higher level of education typically correlates with better learning ability and more frequent mental activity, which helps promote the preservation and connection of neurons in the brain, thus improving cognitive abilities and problem-solving skills [36]. Moreover, patients with a higher level of education generally possess more health awareness and knowledge about their illness, which helps improve their treatment adherence and fosters healthier behaviors [37]. Therefore, healthcare providers should implement more proactive intervention strategies for MHD patients with lower educational levels. Early cognitive function screening, the development of hobbies, and the promotion of cognitive-enhancing activities can effectively stimulate patients’ cognitive potential, enhance brain activity, and delay the progression of cognitive impairment. This approach plays an essential role in optimizing treatment strategies for MHD patients and improving their quality of life.

#### 4.3.2. Hypertension

The results of this study show that patients with hypertension have 23.345 times the risk of developing cognitive impairment compared to those without hypertension, indicating that hypertension is a risk factor for cognitive function decline in MHD patients (OR = 23.345, *p* = 0.007). This finding is consistent with the results of Ko et al. [38], further strengthening the scientific basis for the link between hypertension and cognitive decline. Studies have shown that hypertension can cause various cerebrovascular pathologies, such as arteriosclerosis and vascular spasms, which reduce cerebral blood flow and may lead to lacunar infarctions and small vessel hemorrhages, affecting critical cognitive areas, such as the brain’s white matter and hippocampus. Insufficient blood supply to these areas causes neuronal damage, ultimately leading to cognitive impairment [39]. Thus, healthcare workers should guide patients in regularly monitoring blood pressure and using antihypertensive medications to maintain stable blood pressure, minimizing its harmful effects on cognitive function. Additionally, enhancing patient and family education is crucial to raise awareness about the relationship between blood pressure management and cognitive protection. Encouraging physical activities, such as brisk walking or other aerobic exercises, has been proven to improve cardiovascular health and positively affect cognitive function.

#### 4.3.3. Sleep quality

The results of this study indicate that each point in the PSQI accounts for 15% of the variance in lower cognitive function, suggesting that higher PSQI scores are associated with lower cognitive function scores in MHD patients. This highlights that poor sleep quality is a risk factor for cognitive impairment in MHD patients (OR = 0.852, *p* = 0.034). This finding is consistent with the study by Tian et al. [40]. Poor sleep quality may impair the brain’s ability to clear toxic molecules, such as beta-amyloid and tau protein, that damage neurons and glial cells, leading to cognitive impairment [41]. Additionally, sleep disorders affect the balance of neurotransmitters, especially those closely related to cognitive function, such as acetylcholine and dopamine, further impairing cognitive functions like learning, memory, and judgment [41]. Given the profound impact of sleep quality on brain health and cognitive function, healthcare workers should help patients establish regular sleep routines, create comfortable sleep environments by reducing noise and light interference, and guide them in moderate physical activity to promote relaxation and improve sleep quality. It is also essential to monitor patients’ emotional changes, providing psychological support to alleviate anxiety, depression, and other emotional issues that may affect sleep quality. If necessary, under medical supervision, sleep aids or other treatments for sleep disorders may be considered to improve patients’ sleep quality and enhance cognitive function.

#### 4.3.4. Appetite

The results also show that each point in the VAS accounts for 8% of the variance in higher cognitive function, indicating that higher VAS scores are associated with better cognitive function scores in MHD patients. This demonstrates that better appetite is a protective factor for cognitive function in MHD patients (OR = 1.082, *p* = 0.012). This finding is consistent with the study by Jiang et al. [42]. It has been reported that CKD patients on maintenance dialysis may experience metabolic disorders that can lead to reduced appetite and the occurrence of protein-energy wasting (PEW). Studies have found that 28–54% of maintenance dialysis patients have PEW [43]. This, in turn, can cause malnutrition. The deficiency of essential nutrients, such as vitamins and fatty acids, can exacerbate inflammatory responses in the body, with inflammatory factors penetrating the blood-brain barrier, triggering neuroinflammation and oxidative stress, causing the apoptosis of glial cells and neuronal damage, which leads to cognitive impairment and cerebrovascular damage [44]. Long-term malnutrition also inhibits the regeneration of neurons, disrupts neurotransmitter balance, and damages brain structures, accelerating cognitive decline [45]. Therefore, healthcare workers should closely monitor the nutritional status of MHD patients by regularly assessing key indicators, such as body weight and serum protein levels to promptly identify and address the risk of malnutrition. Individualized nutritional interventions should be developed to ensure patients receive adequate protein, fatty acids, vitamins, and minerals. Additionally, patients should be encouraged to increase physical activity to improve blood circulation and metabolism, boost appetite, and improve nutrient absorption efficiency. Psychological well-being should also be considered, providing necessary counseling to alleviate anxiety and depression, and enhance patient compliance with diet and treatment, thereby promoting cognitive function recovery and improving quality of life.

#### 4.3.5. Family care

Additionally, the findings reveal that each point in the APGSR accounts for 63% of the variance in higher cognitive function, suggesting that higher APGAR scores are associated with better cognitive function scores in MHD patients. This underscores that a higher level of family support is a protective factor for cognitive function (OR = 1.630, *p* = 0.02). Studies have shown that patients who receive higher levels of family care feel more understood, supported, and cared for by their family members, helping to alleviate anxiety, depression, and other negative emotions. Emotional issues, such as anxiety and depression, are common comorbidities in patients with cognitive impairment, and family support can significantly reduce these emotional burdens, thereby maintaining mental health and indirectly promoting the stability or recovery of cognitive function [46]. Further analysis reveals that family members’ companionship and encouragement provide critical motivation for patients to engage in cognitive training and social activities, increasing their willingness and enthusiasm to participate, which stimulates brain function and helps delay cognitive decline [47]. Therefore, healthcare workers should enhance the family support system by strengthening communication with patients’ families and explaining the importance of family care for cognitive function. Family members should be encouraged to spend more time with patients, offering emotional support, listening to their feelings and needs, and making them feel the warmth of the family. Healthcare workers should also guide family members to actively participate in patients’ cognitive rehabilitation training, assisting in a variety of cognitive exercises to promote brain function in a scientific way. Additionally, family members should be attentive to changes in the patient’s emotions, providing timely psychological support and intervention when signs of anxiety or depression arise, to prevent emotional problems from adversely affecting cognitive function. To further enhance family members’ caregiving skills, healthcare workers should provide regular training on relevant caregiving knowledge and skills to ensure that family members can effectively participate in the care process, collectively promoting the recovery of cognitive function and the improvement of quality of life.

### 4.4. The impact of different time points during the dialysis cycle on cognitive function measurement

It is worth noting that different time points during the dialysis cycle significantly affect cognitive function measurements. Previous studies have shown that the dialysis process itself triggers a series of physiological changes, which impact cognitive function differently at various time points. In the early stage of dialysis, abrupt hemodynamic changes, such as rapid decreases or increases in blood volume and fluctuations in blood pressure, may lead to insufficient cerebral perfusion, causing patients to experience symptoms of cognitive decline, such as dizziness and difficulty concentrating. At the same time, the rapid clearance of toxins during dialysis, although beneficial for improving the brain’s microenvironment in the long term, may cause short-term instability in the body’s environment, thus affecting cognitive function. In some patients, cognitive function-related indicators may show significant fluctuations within 1–2 h after the start of dialysis due to changes in blood volume [5]. As dialysis progresses, in the mid-to-late stage of dialysis, although toxin levels continue to decrease, dialysis-related inflammatory responses, electrolyte fluctuations, and other factors still persist. Electrolyte imbalances, such as abnormalities in potassium, sodium, calcium ions, etc., can affect nerve conduction and, in turn, interfere with cognitive function. Furthermore, prolonged dialysis treatments can cause patients to feel fatigued, and this change in physical state may also be reflected in the cognitive function assessment results [18,48,49].

In this study, we chose to assess cognitive function at least 24 h after dialysis, aiming to stabilize the patient’s physical state and minimize the interference of dialysis procedures on cognitive function assessment, thereby providing a more accurate reflection of the patient’s cognitive level in a stable daily state. Although the timing choice in this study is somewhat ­reasonable, to gain a more comprehensive understanding of the dynamic changes in cognitive function throughout the dialysis cycle, future research could consider conducting multiple cognitive function assessments at different time points before and after dialysis (e.g., 1, 6, and 24 h after dialysis). This approach could more clearly depict the dynamic trajectory of cognitive function during the dialysis cycle and provide more precise timing for clinical interventions.

### 4.5. Limitations

This study used a longitudinal research method, combined with the latent class growth model, to analyze the dynamic trajectory of cognitive function changes in MHD patients, identifying two categories: low cognitive function-rapid decline group and high cognitive function-slow decline group. These findings provide new insights into the complexity of cognitive function changes in MHD patients and lay a solid theoretical foundation for developing personalized intervention strategies and optimizing clinical management. The research results also fully confirm the feasibility and practical significance of this study, providing a reference for the next phase of the study. However, this study still has some limitations: (1) This study is a single-center study with a relatively limited sample size, which affects the representativeness and generalizability of the results and may impact the model’s stability. Future research should expand the study scope by conducting multicenter, large-sample studies to increase geographic diversity and improve the generalizability and reliability of the conclusions. (2) Although this study included a series of key variables in the analysis of influencing factors, there is still room for expansion. Future research should aim to build a more comprehensive analytical framework by clinical parameters, incorporating additional laboratory indicators, disease-related factors, family environment variables, and psychosocial factors to fully analyze the factors influencing the latent classes of cognitive function in MHD patients. (3) This study only tracked cognitive function changes in MHD patients for 9 months, capturing short-term trends but not fully reflecting the long-term trajectory of cognitive function changes. Therefore, in the future, we will extend the follow-up period, expand the study scope, and include a wider range of variables to more accurately depict the dynamic changes in cognitive function among MHD patients. Furthermore, we will conduct a more comprehensive investigation of its influencing factors to provide a scientific basis for the development of long-term intervention and management strategies.

## 5. Conclusion

This study, based on the latent class growth model, revealed a linear declining trend in cognitive function among MHD patients. The trajectory of cognitive function changes could be divided into two potential categories: low cognitive function-rapid decline group and high cognitive function-slow decline group. Further exploration indicated that educational level, hypertension, sleep quality, appetite, and family care significantly influence the trajectory of cognitive function changes in MHD patients. Healthcare workers can develop more personalized and precise nursing interventions based on the characteristics of the different latent classes and their influencing factors, effectively delaying or improving cognitive function decline, thereby enhancing patients’ overall quality of life. This approach not only reflects the humanistic care in medical services but also provides new scientific evidence and practical guidance for optimizing the long-term management strategies of MHD patients.

## Data Availability

The data are currently not publicly available due to participant privacy, but they are available from the corresponding author upon reasonable request.
